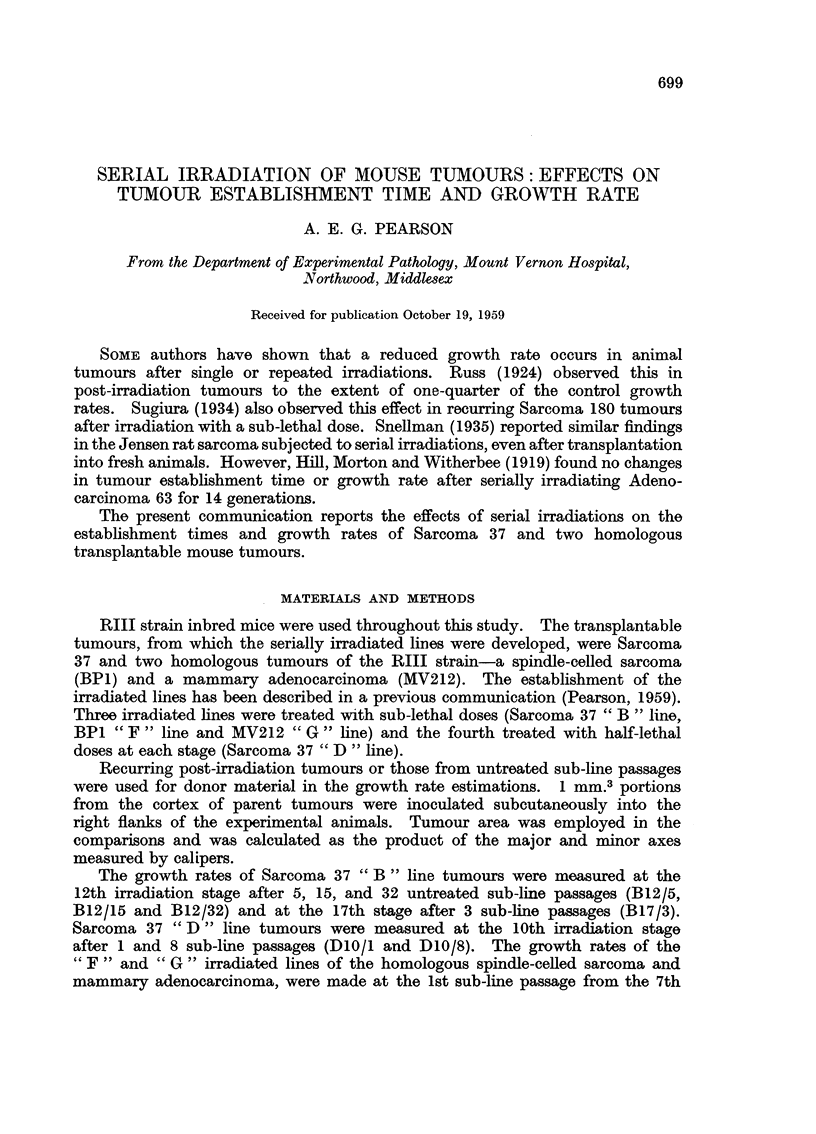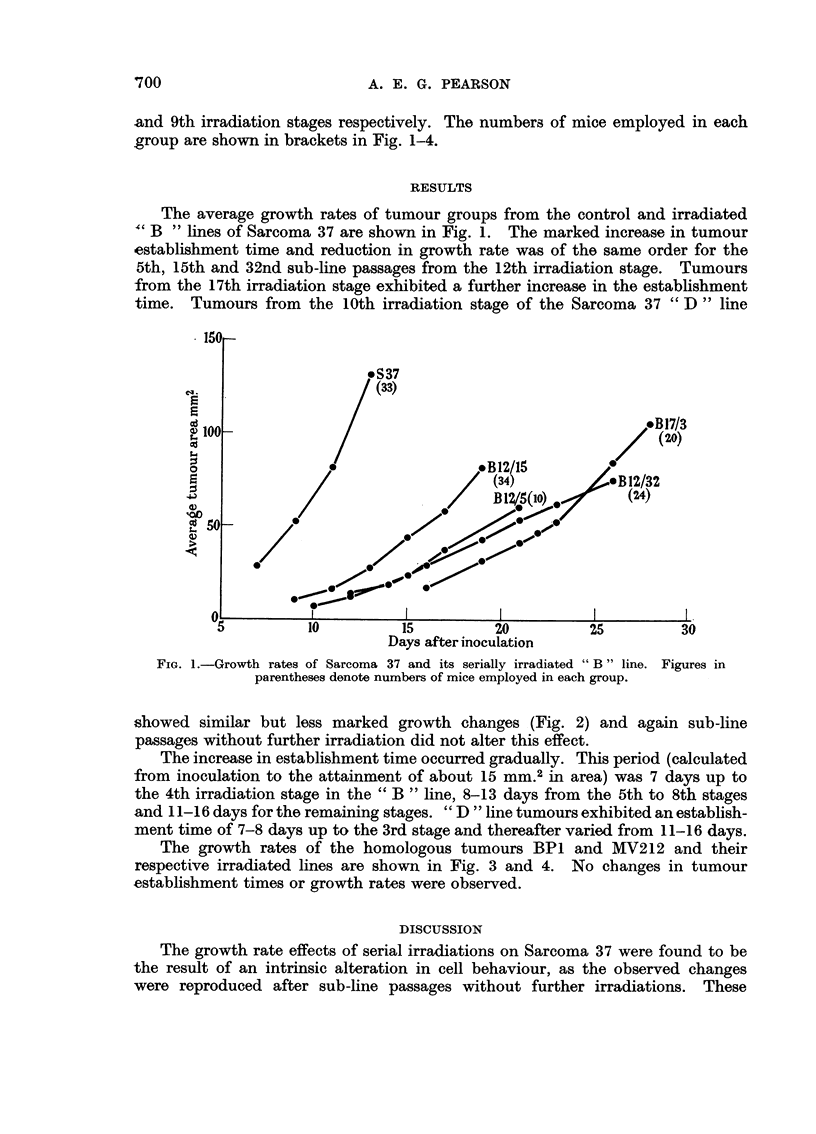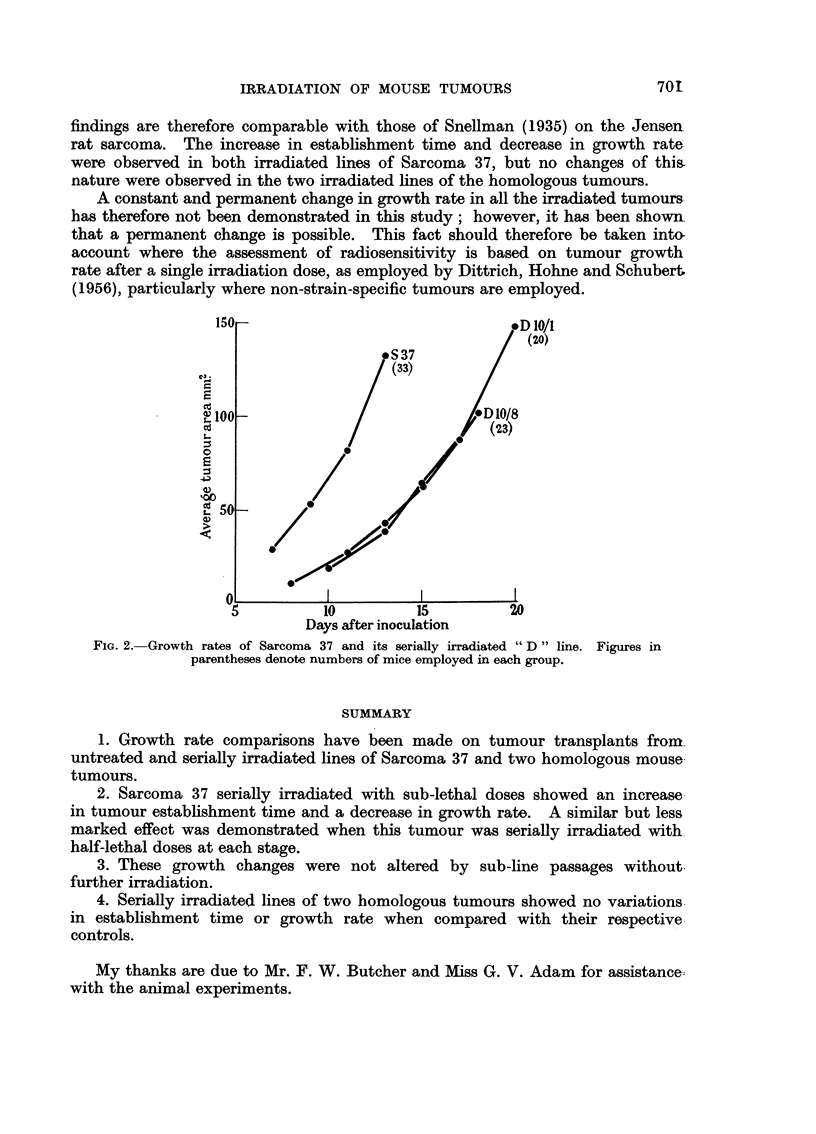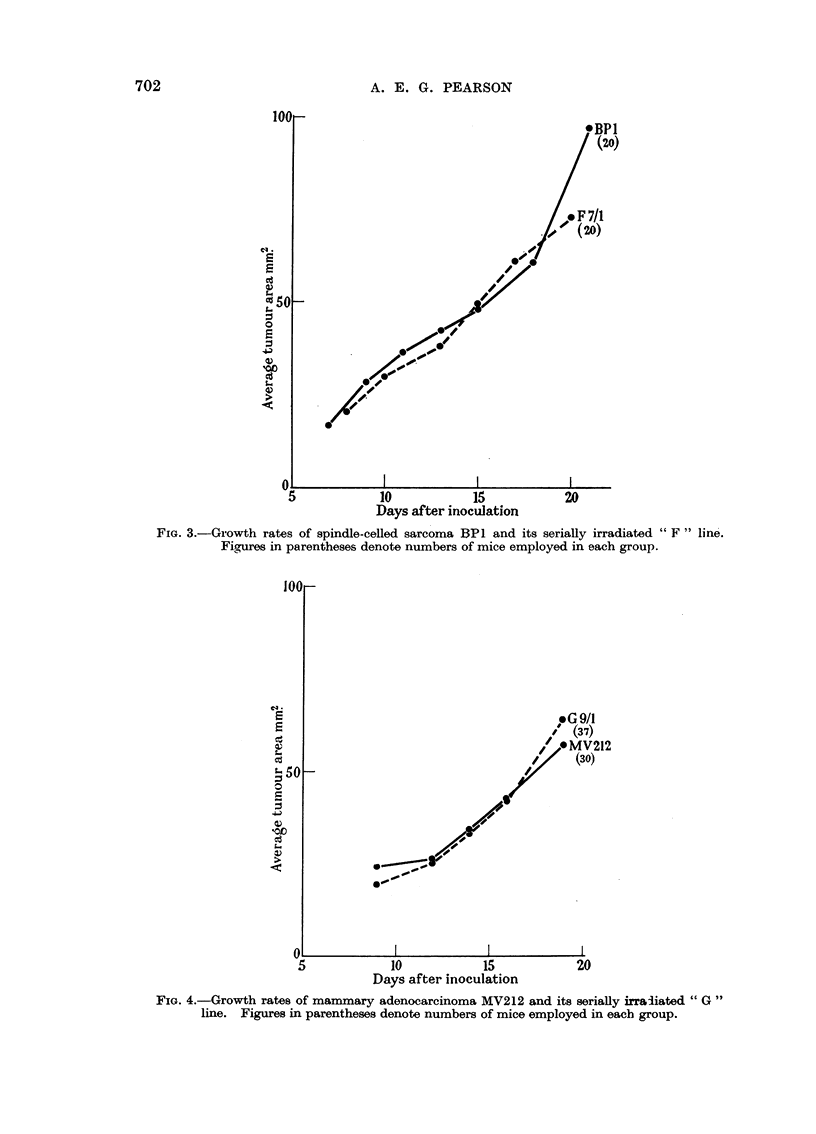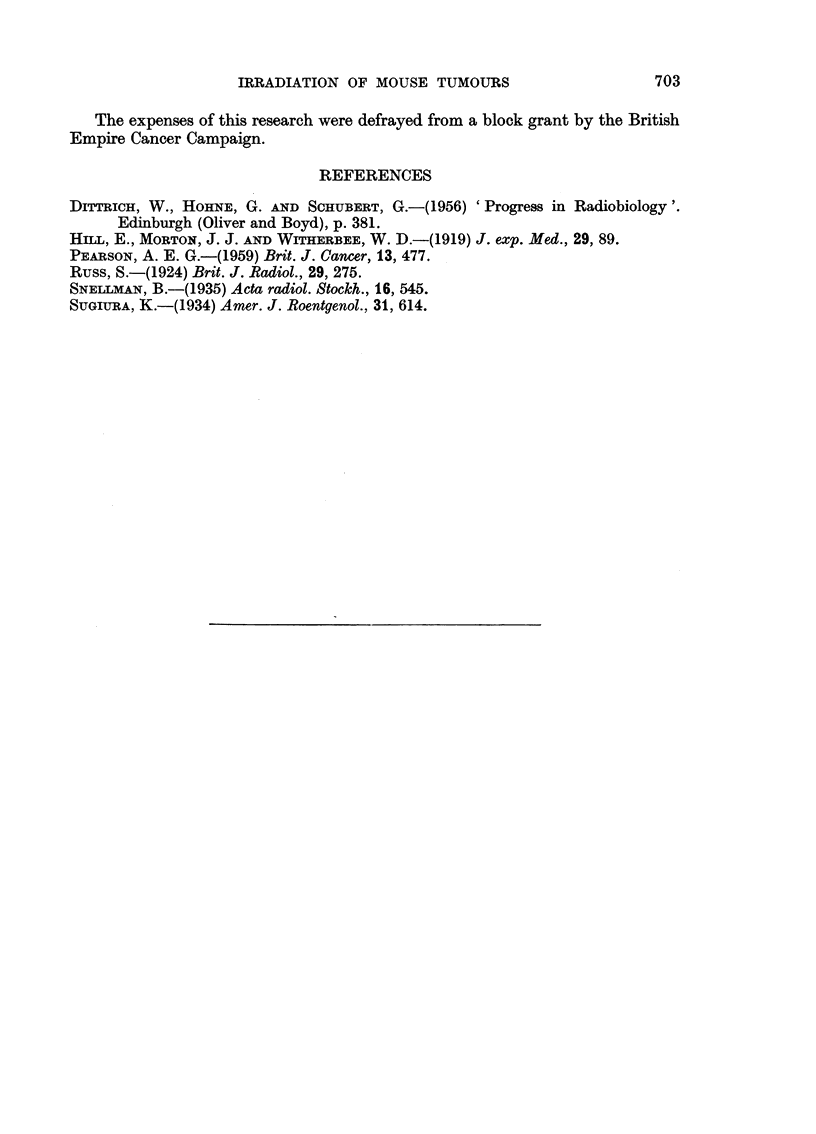# Serial Irradiation of Mouse Tumours: Effects on Tumour Establishment Time and Growth Rate

**DOI:** 10.1038/bjc.1959.78

**Published:** 1959-12

**Authors:** A. E. G. Pearson


### SERIAL IRRADIATION OF MOUSE TUMOURS: EFFECTS ON TUMOUR ESTABLISHMENT TIME AND GROWTH RATE

A. E. G. PEARSON

*From the Department of Experimental Pathology, Mount Vernon Hospital, Northwood, Middlesex*

Received for publication October 19, 1959

SOME authors have shown that a reduced growth rate occurs in animal tumours after single or repeated irradiations. Russ (1924) observed this in post-irradiation tumours to the extent of one-quarter of the control growth rates. Sugiura (1934) also observed this effect in recurring Sarcoma 180 tumours after irradiation with a sub-lethal dose. Snellman (1935) reported similar findings in the Jensen rat sarcoma subjected to serial irradiations, even after transplantation into fresh animals. However, Hill, Morton and Witherbee (1919) found no changes in tumour establishment time or growth rate after serially irradiating Adeno-carcinoma 63 for 14 generations.

The present communication reports the effects of serial irradiations on the establishment times and growth rates of Sarcoma 37 and two homologous transplantable mouse tumours.

### MATERIALS AND METHODS

RIII strain inbred mice were used throughout this study. The transplantable tumours, from which the serially irradiated lines were developed, were Sarcoma 37 and two homologous tumours of the RIII strain—a spindle-celled sarcoma (BP1) and a mammary adenocarcinoma (MV212). The establishment of the irradiated lines has been described in a previous communication (Pearson, 1959). Three irradiated lines were treated with sub-lethal doses (Sarcoma 37 " B " line, BP1 " F " line and MV212 " G " line) and the fourth treated with half-lethal doses at each stage (Sarcoma 37 " D " line).

Recurring post-irradiation tumours or those from untreated sub-line passages were used for donor material in the growth rate estimations. 1 $mm.^3$ portions from the cortex of parent tumours were inoculated subcutaneously into the right flanks of the experimental animals. Tumour area was employed in the comparisons and was calculated as the product of the major and minor axes measured by calipers.

The growth rates of Sarcoma 37 " B " line tumours were measured at the 12th irradiation stage after 5, 15, and 32 untreated sub-line passages (B12/5, B12/15 and B12/32) and at the 17th stage after 3 sub-line passages (B17/3). Sarcoma 37 " D " line tumours were measured at the 10th irradiation stage after 1 and 8 sub-line passages (D10/1 and D10/8). The growth rates of the " F " and " G " irradiated lines of the homologous spindle-celled sarcoma and mammary adenocarcinoma, were made at the 1st sub-line passage from the 7th and 9th irradiation stages respectively. The numbers of mice employed in each group are shown in brackets in Fig. 1–4.

### RESULTS

The average growth rates of tumour groups from the control and irradiated " B " lines of Sarcoma 37 are shown in Fig. 1. The marked increase in tumour establishment time and reduction in growth rate was of the same order for the 5th, 15th and 32nd sub-line passages from the 12th irradiation stage. Tumours from the 17th irradiation stage exhibited a further increase in the establishment time. Tumours from the 10th irradiation stage of the Sarcoma 37 " D " line

FIG. 1.—Growth rates of Sarcoma 37 and its serially irradiated " B " line. Figures in parentheses denote numbers of mice employed in each group.

showed similar but less marked growth changes (Fig. 2) and again sub-line passages without further irradiation did not alter this effect.

The increase in establishment time occurred gradually. This period (calculated from inoculation to the attainment of about 15 mm.² in area) was 7 days up to the 4th irradiation stage in the " B " line, 8–13 days from the 5th to 8th stages and 11–16 days for the remaining stages. " D " line tumours exhibited an establishment time of 7–8 days up to the 3rd stage and thereafter varied from 11–16 days.

The growth rates of the homologous tumours BP1 and MV212 and their respective irradiated lines are shown in Fig. 3 and 4. No changes in tumour establishment times or growth rates were observed.

### DISCUSSION

The growth rate effects of serial irradiations on Sarcoma 37 were found to be the result of an intrinsic alteration in cell behaviour, as the observed changes were reproduced after sub-line passages without further irradiations. These findings are therefore comparable with those of Snellman (1935) on the Jensen rat sarcoma. The increase in establishment time and decrease in growth rate were observed in both irradiated lines of Sarcoma 37, but no changes of this nature were observed in the two irradiated lines of the homologous tumours.

A constant and permanent change in growth rate in all the irradiated tumours has therefore not been demonstrated in this study; however, it has been shown that a permanent change is possible. This fact should therefore be taken into account where the assessment of radiosensitivity is based on tumour growth rate after a single irradiation dose, as employed by Dittrich, Hohne and Schubert (1956), particularly where non-strain-specific tumours are employed.

FIG. 2.—Growth rates of Sarcoma 37 and its serially irradiated "D" line. Figures in parentheses denote numbers of mice employed in each group.

SUMMARY

1. Growth rate comparisons have been made on tumour transplants from untreated and serially irradiated lines of Sarcoma 37 and two homologous mouse tumours.

2. Sarcoma 37 serially irradiated with sub-lethal doses showed an increase in tumour establishment time and a decrease in growth rate. A similar but less marked effect was demonstrated when this tumour was serially irradiated with half-lethal doses at each stage.

3. These growth changes were not altered by sub-line passages without further irradiation.

4. Serially irradiated lines of two homologous tumours showed no variations in establishment time or growth rate when compared with their respective controls.

My thanks are due to Mr. F. W. Butcher and Miss G. V. Adam for assistance with the animal experiments.

FIG. 3.—Growth rates of spindle-celled sarcoma BP1 and its serially irradiated " F " line. Figures in parentheses denote numbers of mice employed in each group.

FIG. 4.—Growth rates of mammary adenocarcinoma MV212 and its serially irradiated " G " line. Figures in parentheses denote numbers of mice employed in each group.

The expenses of this research were defrayed from a block grant by the British Empire Cancer Campaign.

### REFERENCES

DITTRICH, W., HOHNE, G. AND SCHUBERT, G.—(1956) 'Progress in Radiobiology'. Edinburgh (Oliver and Boyd), p. 381.
HILL, E., MORTON, J. J. AND WITHERBEE, W. D.—(1919) *J. exp. Med.*, **29**, 89.
PEARSON, A. E. G.—(1959) *Brit. J. Cancer*, **13**, 477.
RUSS, S.—(1924) *Brit. J. Radiol.*, **29**, 275.
SNELLMAN, B.—(1935) *Acta radiol. Stockh.*, **16**, 545.
SUGIURA, K.—(1934) *Amer. J. Roentgenol.*, **31**, 614.